# Circadian Regulation of Vitamin D Target Genes Reveals a Network Shaped by Individual Responsiveness

**DOI:** 10.3390/nu17071204

**Published:** 2025-03-29

**Authors:** Parcival Maissan, Carsten Carlberg

**Affiliations:** Institute of Animal Reproduction and Food Research, Polish Academy of Sciences, 10-683 Olsztyn, Poland; p.maissan@pan.olsztyn.pl

**Keywords:** vitamin D, circadian gene regulation, vitamin D target genes, differential gene expression, transcriptome, vitamin D receptor

## Abstract

Background: In humans, vitamin D_3_ synthesis follows a day–night rhythm due to its UV-B-dependent production. Results: As part of the VitDHiD intervention study, we identified 87 in vivo vitamin D target genes with circadian expression patterns in immune cells, forming a regulatory network centered on transcription factors and membrane receptors. These genes exhibit a narrow basal expression range, with 80% downregulated upon vitamin D_3_ supplementation. Clustering analysis revealed six distinct gene groups, with the two most prominent clusters driven by the transcription factor CSRNP1 (cysteine- and serine-rich nuclear protein 1) and GAS7 (growth arrest-specific 7), a known differentiation inducer. Among the 25 VitDHiD study participants, we identified two subgroups distinguished by significant differences in the responsiveness of 14 in vivo vitamin D target genes. These genes encode transcription factors like CSRNP1, as well as metabolic enzymes and transporters, including NAMPT (nicotinamide phosphoribosyltransferase), PFKFB3 (6-phosphofructo-2-kinase/fructose-2,6-bisphosphatase 3), and SLC2A3 (solute carrier family 2 member 3). Notably, all 14 genes possess a vitamin D receptor-binding enhancer within a reasonable distance of their transcription start site. Conclusions: These findings highlight a novel link between vitamin D signaling and circadian gene regulation, with potential implications for personalized supplementation strategies.

## 1. Introduction

Vitamin D_3_ is traditionally classified as a vitamin, yet humans can endogenously synthesize it through UV-B-induced conversion of 7-dehydrocholesterol in the skin [1,2]. However, approximately 50,000 years ago, the migration of *Homo sapiens* from the consistently sunlit regions of Africa to areas in Europe and Asia with significantly lower UV exposure transformed this molecule into a vital nutrient for populations in those latitudes [3,4,5,6].

The role of vitamin D in calcium regulation has been critical since vertebrate species transitioned from aquatic to terrestrial environments around 380 million years ago, requiring stable skeletal structures [7]. Consequently, a hallmark of vitamin D deficiency is the development of bone abnormalities [8]. Beyond skeletal health, vitamin D is also integral to the regulation of both innate and adaptive immune systems [9]. Maintaining sufficient vitamin D levels has been associated with reduced risks of various conditions, including musculoskeletal disorders such as osteoporosis [10] and sarcopenia [11], autoimmune diseases like multiple sclerosis [12], and certain cancers, such as colorectal cancer [13]. Nevertheless, large-scale interventional studies, involving thousands of participants over periods of up to five years, have struggled to provide statistically significant evidence for primary health benefits of vitamin D_3_ supplementation [14,15]. Despite lack of health benefits for the general population, in the DO-HEALTH study, which focused on healthy elderly individuals, participants aged 70–74 years had significantly lower infection rates, suggesting a potential effect of vitamin D_3_ supplementation on the immune system [16].

The molecular mechanisms of vitamin D are centered on its nuclear receptor, VDR (vitamin D receptor), which exhibits an exceptionally high binding affinity (K_D_ = 0.1 nM) for the active vitamin D metabolite, 1α,25-dihydroxyvitamin D_3_ (1,25(OH)_2_D_3_) [17,18]. In the endocrine system, this active form is primarily synthesized in the kidneys from 25-hydroxyvitamin D_3_; however, it can also be produced locally in immune and skin cells for autocrine and paracrine signaling [19]. As a transcription factor, VDR governs all genomic actions of vitamin D, with 1,25(OH)_2_D_3_ being its sole physiological activator [20]. VDR is widely expressed across most tissues, with the exception of the brain, and regulates hundreds of genes in a tissue- and individual-specific manner [15]. This variability in gene responsiveness forms the basis for the vitamin D response index, which classifies individuals as high, mid, or low responders to vitamin D supplementation [21]. This heterogeneity in response might contribute to the difficulty of finding health benefits for vitamin D_3_ supplementation in studies targeting the general population.

To explore the molecular effects of vitamin D in vivo under healthy conditions, studies such as VitDbol (NCT02063334) [22,23] and VitDHiD (NCT03537027) [24,25] were conducted with cohorts in Kuopio, Finland, who received in total 80,000 IU of vitamin D_3_ administered in two equal doses along with breakfast and lunch, which is equivalent to a monthly dose. The *PER1* (period circadian regulator 1) gene, a key regulator of circadian rhythms, emerged as a prominent in vivo vitamin D target in the VitDHiD study. Given that vitamin D_3_ synthesis in the skin naturally follows a day–night rhythm, we sought to explore whether vitamin D also influences the expression of circadian genes. While the impact of vitamin D on immune function is well established, its potential role in modulating circadian gene expression remains largely unexplored. This study seeks to bridge this gap by identifying circadian vitamin D target genes and exploring interindividual variability in responsiveness.

This study presents a secondary analysis of the VitDHiD trial, focusing on 7665 genes with stable expression profiles in peripheral blood mononuclear cells (PBMCs) from 25 study participants. Of the 361 in vivo vitamin D target genes, 87 exhibit a circadian expression pattern. This suggests that fine-tuning the circadian rhythm of gene expression is a previously underexplored physiological function of vitamin D.

## 2. Materials and Methods

### 2.1. Transcriptome Data Sources

The VitDHiD trial investigated transcriptome changes in PBMCs from 25 healthy individuals (age 20–54 years, 12 females and 13 males, body mass index 21.4–25.6) supplemented with a single vitamin D_3_ bolus (80,000 IU) over one day [25]. Our analysis focused on 7665 genes with stable expression levels exceeding 10 counts per million (CPM) across all participants (Table S1). Among these, 361 genes exhibited significant changes in expression (FDR (false discovery rate) ≤ 0.05) when comparing baseline levels to 24 h post-supplementation with vitamin D_3_. A comprehensive literature and database search for transcriptome datasets describing circadian gene expression in vivo in human blood cells identified only one relevant study. This report analyzed the circadian expression profile of the whole blood transcriptome of 26 participants undergoing a 1-week sleep restriction study controlled with 1-week of sufficient sleep; after each condition, blood was sampled every 3 h for 30 h to detect genes with a circadian rhythm. We used the results from the control group in our analysis [26]. Additionally, seasonally expressed genes were identified based on a 4-year profiling of PBMCs from 105 individuals in California [27]. To ensure consistency and adherence to current nomenclature, gene symbols across all datasets were updated using either the HGNC Multi-Symbol Checker [28] (www.genenames.org/tools/multi-symbol-checker, accessed on 22 November 2024) when only gene symbols were available or g:Profiler’s [29] (https://biit.cs.ut.ee/gprofiler/convert, accessed on 22 November 2024) gene ID conversion for ENSG identifiers. Outdated symbols were replaced with their current approved equivalents, while aliases were excluded to avoid potential errors caused by genes sharing similar aliases, which could introduce false overlap during analysis. If no matching gene symbol was available, or if it was withdrawn, the entry was removed from the list. After this update, the circadian gene list contained 1461 genes, while the seasonal gene list included 757 genes. Within the 7665 commonly expressed genes, 873 and 363 members of these genes were found to be expressed (Table S1).

### 2.2. Differential Gene Expression Analysis

The raw count data from the VitDHiD study [25] were used in this analysis. Differential gene expression analysis was conducted in R (version 4.4.1) on MacOS 15 (Sequoia, Menlo Park, CA, USA) using EdgeR [30] (version 4.4.2). The analysis focused on 19,824 expressed protein-coding genes to minimize transcriptional noise from non-coding or lowly expressed genes. Read counts were normalized to CPM to account for differences in library size. To remove genes that have insufficient read counts for reliable statistical inference, genes with very low expression were filtered out using the *FilterByExpr()* function and removing all genes with a CPM ≤ 10. After filtering, library sizes were recomputed, and trimmed mean of M-values (TMM) normalization was applied. Differential expression between clusters was assessed using the generalized linear model (GLM) quasi-likelihood pipeline available in EdgeR, as described by its developer [31].

### 2.3. Characterization of Target Genes

The STRING database (http://string-db.org, accessed on 22 January 2025, version 12.0) [32] was employed to confirm and visualize the physical and functional interactions among circadian and vitamin D target genes. In the resulting network, each node represents a protein, while edges between nodes indicate associations derived from experimental data, computational predictions, and literature sources. Functional annotations of the genes, with a focus on their primary biological roles, were obtained from resources such as the Human Protein Atlas (www.proteinatlas.org, accessed on 14 December 2024) [33] and GeneCards (www.genecards.org, accessed on 14 December 2024) [34].

To further investigate gene relationships, hierarchical clustering was performed on the Pearson correlation matrix using correlation distance as the metric and complete linkage as the clustering method; the optimal amount of correlation clusters was determined by the silhouette method. For the heatmap visualization of the log_2_FC (fold change) across individuals, *k-means* clustering was applied to partition individuals into two groups, while hierarchical clustering was performed on the genes. The resulting correlation matrix and clustering results were visualized as a heatmap using the pheatmap R package (version 1.0.12). GeneMANIA (https://genemania.org, accessed on 2 March 2025) [35] was used to see which genes had previously reported coexpressions.

### 2.4. Analysis of Genomic Regions of Vitamin D Target Genes

To investigate regulatory elements in key in vivo vitamin D target genes, genomic regions were analyzed to identify VDR-binding enhancers and TSS (transcription start site) regions. This analysis leveraged epigenome-wide data from THP-1 cells stimulated with either 10 nM 1,25(OH)_2_D_3_ or a solvent control (0.1% ethanol) for 2 and 24 h. ChIP-seq (chromatin immunoprecipitation sequencing) datasets [36] were used to pinpoint VDR-binding sites, while FAIRE-seq (formaldehyde-assisted isolation of regulatory elements followed by sequencing) data [37] provided insights into chromatin accessibility, aiding in the identification of active regulatory regions. Visualization of these datasets was performed using the IGV browser [38], allowing the examination of VDR-binding enhancers and TSS regions within accessible chromatin that respond to 1,25(OH)_2_D_3_. These regulatory elements were assessed within TAD (topologically associating domain) regions of vitamin D target genes. While a broad genomic window of ±1 Mb around each target gene’s TSS was initially screened, only the most relevant regions are reported in this analysis.

### 2.5. Statistical Tests

To assess the significance of potential overlap among the VitDHiD (361 genes), circadian (873 genes), and seasonal (363 genes) gene sets, a hypergeometric distribution test was performed using R. Additionally, to evaluate statistically significant correlations (*p* ≤ 0.05) in the expression of circadian and vitamin D target genes, a Pearson correlation analysis was conducted, applying the Benjamini–Hochberg correction for multiple testing. To identify significant differences in gene expression between the two clusters of individuals, a *t*-test was performed, followed by the Benjamini–Hochberg correction for multiple testing.

## 3. Results

### 3.1. Circadian Expression Profile of In Vivo Vitamin D Target Genes

A transcriptomic dataset from the vitamin D_3_ intervention study VitDHiD, which involved 25 healthy individuals supplemented with 80,000 IU of vitamin D_3_ over 24 h, was analyzed as a reference for identifying in vivo vitamin D target genes in PBMCs [25]. After filtering for genes with a minimum expression of CPM ≥ 10 across all individuals, 7665 genes were classified as commonly expressed. Of these, 361 genes were significantly responsive to vitamin D_3_ supplementation (FDR ≤ 0.05) (Table S1).

**Figure 1:**
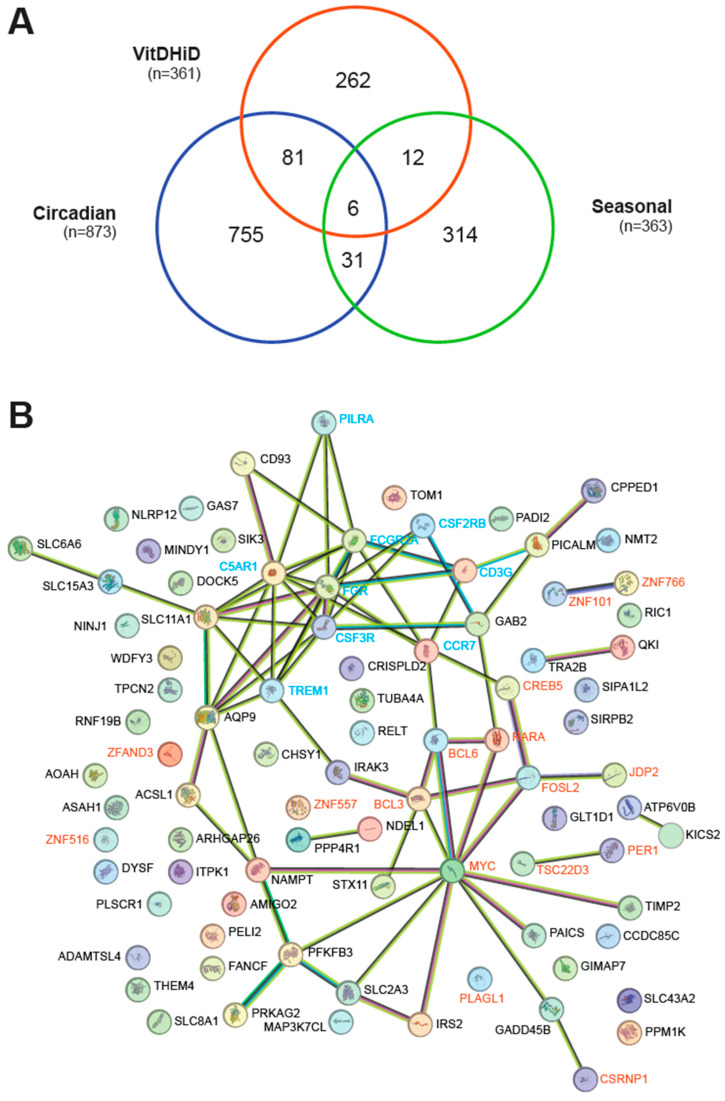
Circadian expression profile of in vivo vitamin D target genes. (**A**) A Venn diagram illustrating the overlap between in vivo vitamin D target genes, circadian genes, and seasonal genes. (**B**) Gene/protein interaction network for the 87 circadian genes identified as in vivo vitamin D targets, based on STRING database analysis. Nodes represent proteins, while edges denote both functional and physical associations. Edge thickness indicates the strength of supporting data. Transcription factors and regulators are highlighted in red, while membrane receptor genes are shown in blue.

Among the 7665 commonly expressed genes, 873 were found in the curated and updated list of 1462 circadian genes in the whole blood transcriptome of 26 participants in the control arm of a sleep study [26]. Additionally, 363 of 757 seasonally expressed genes, identified from the curated and updated PBMC transcriptome of 105 individuals [27], overlapped with the 7665 genes (Table S1). Notably, 87 and 18 of the 361 vitamin D target genes exhibited circadian and seasonal expression patterns, respectively (Figure 1A). Furthermore, the list of circadian and seasonal genes overlapped in 37 genes, 6 of which were also vitamin D targets: *CREB5* (CAMP responsive element binding protein 5), *DYSF* (dysferlin), *PER1*, *SIK3* (SIK family kinase 3), *SLC8A1*, and *WDFY3* (WD repeat and FYVE domain containing 3).

Proteins encoded by the 87 in vivo vitamin D target genes exhibiting circadian behavior were analyzed using the STRING database [32]. Approximately half of these proteins are part of a network centered around the transcription factors BCL6 (BCL6 transcription coactivator), CSRNP1, CREB5, FOSL2 (FOS-like 2, AP1 transcription factor subunit), JDP2 (Jun dimerization protein 2), MYC (MYC proto-oncogene, BHLH transcription factor), and RARA (retinoic acid receptor alpha) as well as the transcriptional cofactor BCL3 (Figure 1B). Additionally, the transcriptional cofactors PER1 and TSC22D3 (TSC22 domain family member 3), along with the transcription factors ZNF101 (zinc finger protein 101) and ZNF766, were also found to be connected within this network. Another network centered on the membrane receptor proteins C5AR1 (complement C5a receptor 1), CCR7 (C-C motif chemokine receptor 7), CD3G (CD3 gamma subunit of T-cell receptor complex), CSF2RB (colony stimulating factor 2 receptor subunit beta), CSF3R (colony stimulating factor 3 receptor), FCGR2A (Fc gamma receptor IIa), FGR (FGR proto-oncogene, Src family tyrosine kinase), PILRA (paired immunoglobin-like type 2 receptor alpha), and TREM1 (triggering receptor expressed on myeloid cells 1). In contrast, the analysis of the 18 proteins encoded by vitamin D target genes that exhibit seasonal variation revealed no convincing network using STRING. Only two transcription factor proteins, CREB5 and KLF11 (KLF transcription factor 11), showed a connection (Figure S1). Furthermore, a hypergeometric test indicated that the identification of 18 seasonal genes out of 363 may have occurred by chance (*p* = 0.4233), whereas the 87 vitamin D target genes among the 873 circadian genes were highly significant (*p* = 1.1976 × 10^−12^). Interestingly, 34 of the 87 in vivo vitamin D target genes were also identified in recently published in vitro studies, in which human PBMCs had been stimulated for 24 h with 1,25(OH)_2_D_3_ [24,39,40] (Table S1). These genes include *ADAMTSL4* (ADAMTS-like 4), *AQP9* (aquaporin 9), *BCL3*, *C5AR1*, *CD93* (CD93 molecule), *CPPED1* (calcineurin-like phosphoesterase domain containing 1), *CREB5*, *CRISPLD2* (cysteine-rich secretory protein LCCL domain containing 2), *CSF2RB*, *CSF3R*, *DYSF*, *FCGR2A*, *FOSL2*, *GAS7*, *IRAK3* (interleukin 1 receptor-associated kinase 3), *JDP2*, *MAP3K7CL* (MAP3K7 C-terminal like), *NAMPT*, *NLRP12* (NLR family pyrin domain containing 12), *PADI2* (peptidyl arginine deiminase 2), *PILRA*, *PLAGL1*, *RNF19B* (ring finger protein 19B), *SIPA1L2* (signal-induced proliferation-associated 1 like 2), *SIRPB2* (signal regulatory protein beta 2), *SLC11A1*, *SLC15A3*, *SLC43A2*, *SLC8A1*, STX11 (syntaxin 11), *TIMP2* (TIMP metallopeptidase inhibitor 2), *TREM1*, *WDFY3*, and *ZNF516*.

In summary, 87 in vivo vitamin D target genes display a circadian rhythm. These target genes encode proteins that form a network, with transcription factors and membrane receptors at its core.

### 3.2. Characterization of Vitamin D Targets with Circadian Behavior

The 87 in vivo vitamin D target genes exhibiting circadian expression profiles demonstrate a rather narrow range of basal expression levels, with *AMIGO2* (adhesion molecule with Ig-like domain 2) being only 35-fold less expressed than *TSC22D3* (Figure S2A, Table S1). Of these genes, only 17 are upregulated by vitamin D_3_ supplementation, with *MAP3K7CL* showing the highest induction, while 70 are downregulated, with *JDP2* being the most repressed (Figure S2B).

**Figure 2:**
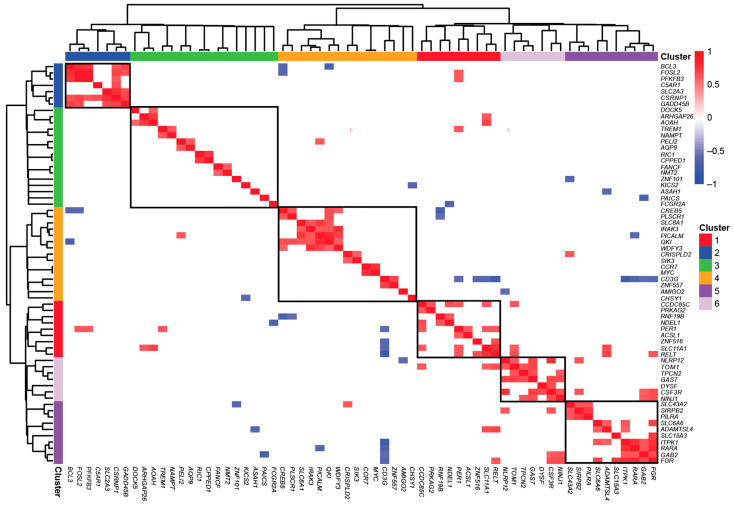
Characterization of vitamin D targets with circadian behavior. Cluster analysis on the Pearson correlation of the 87 circadian genes identified as in vivo vitamin D targets revealed 6 distinct gene clusters.

Hierarchical clustering of the 87 vitamin D target genes for coexpression identified six distinct gene clusters, each comprising 7 to 16 members with positively correlated expression patterns (Pearson correlation *p*_adj_ ≤ 0.05) (Figure 2). Among these, cluster 1 is the most densely connected. The core regulatory hub of this cluster appears to be the *CSRNP1* gene encoding for a transcription factor, whose expression strongly correlates with that of the transcription (co)factors BCL3 and FOSL2, the innate immune receptor C5AR1, the carbohydrate metabolism regulators PFKFB3 and SLC2A3, and the cellular growth and apoptosis regulator GADD45B (growth arrest and DNA damage inducible beta). Cluster 5, in contrast, exhibits a different functional profile. This cluster is primarily driven by the *GAS7* gene encoding a differentiation-promoting protein, whose expression correlates with the cytokine receptor CSF3R, the adhesion protein NINJ1 (ninjurin 1), the ion channel TPCN2 (two-pore segment channel 2), the calcium ion sensor DYSF, the cellular trafficking regulator TOM1 (target of Myb1 membrane trafficking protein), and the inflammation-modulating protein NLRP12. The coexpression results for cluster 1, as verified by GeneMANIA, indicate that *CSRNP1* is coexpressed with all genes except *C5AR1*. However, *C5AR1* is indirectly linked to *CSRNP1* via *SLC2A3* and *GADD45B*. The *GAS7* gene does not exhibit any connections with other genes, whereas *CSF3R* is coexpressed with the genes *TOM1*, *NINJ1*, *DYSF*, and *NLRP12*. Additionally, the genes in cluster 1 are represented in the STRING network, where each gene has at least one connection, except for *C5AR1*, in contrast to cluster 5, where no connections are observed.

Taken together, the 87 circadian genes display a relatively narrow range of basal expression, with 80% being downregulated by vitamin D_3_ supplementation. Among the 6 identified gene clusters, cluster 1 and cluster 5 stand out, driven respectively by the transcription factor CSRNP1 and the differentiation inducer GAS7, emphasizing their distinct functional roles.

### 3.3. Individual-Specific Responses of Circadian Vitamin D Target Genes

**Figure 3:**
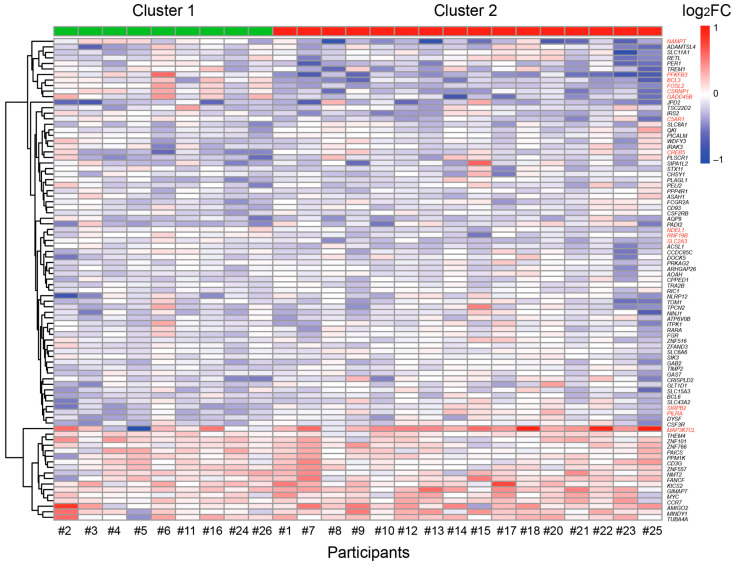
Individual-specific responses of circadian vitamin D target genes. Hierarchical clustering is based on the log_2_FC of individual-specific responses of 87 circadian vitamin D target genes. *K-means* clustering suggested a distinction among the 25 VitDHiD participants, classifying them into two groups (Cluster 1 and Cluster 2). Genes driving the significant differences between these groups are highlighted in red.

Expression analysis of 87 in vivo circadian vitamin D target genes before and after vitamin D_3_ supplementation distinguished the 25 participants of the VitDHiD trial into two groups (Figure 3). Group 1, consisting of 9 individuals, showed significantly higher expression (*p*_adj_ ≤ 0.05) in 14 genes compared to the 16 participants in group 2. These genes were *NAMPT*, *PFKFB3*, *BCL3*, *FOSL2*, *CSRNP2*, *GADD45B*, *C5AR1*, *CREB5*, *NDEL1* (NudE neurodevelopment protein 1 like 1), *RNF19B*, *SLC2A3*, *SIRPB2*, *PILRA*, and *MAP3K7CL*. Differential gene expression analysis revealed no significant differences in gene expression across these clusters prior to vitamin D_3_ supplementation. However, after supplementation, four genes (*CSRNP2*, *FOSL2*, *NDEL1*, and *PFKFB3*) were found to be differentially expressed between the two groups (FDR ≤ 0.05, Table S2).

**Figure 4:**
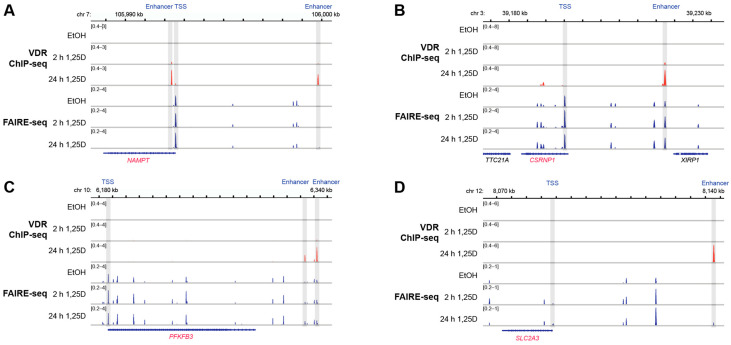
VDR-binding enhancers in the genomic regions of four vitamin D target genes. The IGV browser was used to visualize ChIP-seq results for VDR (red) and FAIRE-seq data (blue) from THP-1 cells treated with solvent (0 h) or 1,25(OH)_2_D_3_ (1,25D) for 2 and 24 h. The genomic region of the vitamin D target genes *NAMPT* (**A**), *CSRNP1* (**B**), *PFKFB3* (**C**) and *SLC2A3* (**D**) are displayed. TSS regions and VDR-binding enhancers are shaded in grey. Peak tracks represent merged data from three biological replicates. Gene structures are depicted in blue, with vitamin D target genes highlighted in red. While genomic regions spanning 1 Mb upstream and downstream of each gene’s TSS were analyzed, only areas relevant to 1,25(OH)_2_D_3_-dependent regulation are shown.

The genomic regions within ±1 Mb of the TSS of these in total 14 in vivo vitamin D target genes were screened for experimentally validated VDR binding sites in accessible chromatin. This analysis utilized VDR ChIP-seq and FAIRE-seq datasets from THP-1 cells treated with either solvent or 10 nM 1,25(OH)_2_D_3_ for 2 and 24 h. At least one VDR-binding enhancer was identified for each of the 14 genes at varying distances from the TSS: 2 kb (*NAMPT*), 6 kb (*BCL3*), 14 kb (*C5AR1*), 16 kb (*RNF19B*), 27 kb (*CSRNP2*), 49 kb (*FOSL2*), 54 kb (*SLC2A3*), 55 kb (*MAP3K7CL*), 69 kb (*GADD45B*), 76 kb (*SIRPB2*), 140 kb (*NDEL1*), 146 kb (*PFKFB3*), 165 kb (*PILRA*), and 455 kb (*CREB5*) (Figure 4 and Figure S3). These enhancers were located within a range that suggests their potential role in regulating gene expression.

In summary, based on the expression of 14 out of 87 circadian in vivo vitamin D target genes, the 25 VitDHiD participants can be categorized into two groups of 9 and 16 individuals. Notably, all 14 genes possess VDR-binding enhancers within 2 to 455 kb of their TSS, suggesting a role for VDR-mediated regulation in the observed differential expression patterns.

## 4. Discussion

This study provides new insights into the circadian expression of vitamin D target genes in vivo, demonstrating a significant overlap between genes responsive to vitamin D_3_ supplementation and those exhibiting circadian rhythmicity. Our findings emphasize the complexity of vitamin D-mediated transcriptional regulation, particularly within PBMCs of healthy individuals. Using transcriptomic data from the VitDHiD vitamin D intervention study, we identified 87 in vivo vitamin D target genes exhibiting circadian expression patterns. Network analysis revealed that the proteins encoded by these 87 circadian vitamin D target genes are functionally interconnected, with transcription factors (e.g., BCL6, CREB5, FOSL2, JDP2, MYC, RARA) and membrane receptors (e.g., C5AR1, CCR7, CD3G, CSF2RB, CSF3R, FCGR2A, FGR, PILRA, TREM1) forming core regulatory hubs. This suggests that vitamin D signaling influences key transcriptional regulators and immune-related pathways in a time-dependent manner. The rhythmic nature of the immune system has been widely reported [41,42], encompassing cell-type composition, both innate and adaptive immune responses, and immune cell functions such as metabolism, migration, and phagocytic activity. Additionally, previous studies have demonstrated that measuring key clock genes in PBMCs allows individuals to be categorized into two chronotypes like morning types (“larks”) and evening types (“owls”), feeling more alert and productive in the morning or evening, respectively [43]. However, due to the design of the VitDHiD study, it is not possible to determine whether the observed clustering is attributable to chronotype differences. Regardless, the finding that vitamin D_3_ supplementation can stratify individuals into two distinct groups of 9 and 16 members, respectively, in a circadian manner is a noteworthy observation.

In contrast, seasonally regulated vitamin D target genes did not display a comparable functional network, reinforcing a stronger association between vitamin D and circadian biology rather than seasonal variation. Although seasonal changes, except close to the equator, are typically characterized by fluctuations in mean ambient temperature and sunlight hours [44], which would be expected to influence circadian rhythms, the overlap between circadian and seasonal genes remains surprisingly limited. Given that circadian entrainment primarily depends on light, with contributions from temperature and other factors, this limited overlap is intriguing from an evolutionary perspective. Modern humans (*Homo sapiens*) evolved in East Africa, where they were consistently exposed to sufficient UV-B radiation year-round, ensuring stable vitamin D_3_ synthesis and adaptation to a regular day–night cycle for over 200,000 years [45]. In contrast, populations migrating beyond 37° N only began experiencing seasonal variations in sunlight exposure and endogenous vitamin D_3_ production approximately 45,000 years ago [46]. Thus, there has been significantly less evolutionary time to adapt to seasonal variations compared to circadian rhythms.

Notably, the circadian vitamin D target genes also clustered into six coexpression groups, with two clusters, *CSRNP1*-driven and *GAS7*-driven, standing out due to their distinct regulatory functions. The *CSRNP1* cluster includes genes involved in transcriptional regulation and metabolism [47]. In this cluster, *C5AR1*, which is as of now underreported in its relations to the other genes in the cluster, is emerging as a driver of complement-mediated regulation of immunometabolism through mitochondrial activity [48]. Given that *CSRNP1* appears to drive this cluster, future research could explore whether *CSRNP1* directly influences immunometabolism through transcriptional regulation. The *GAS7* cluster is particularly intriguing, as the association among these genes appears underreported. However, when analyzing the functions of its constituents, it emerges as a promising target for further investigation. The GAS7 protein itself regulates actin assembly and membrane outgrowth [49], while TOM1, DYSF, and TPCN2 are linked to lysosomal function [50,51]. *CSF3R*, the only gene in the cluster with reported coexpression with all other genes, is well known for its central role in granulocyte differentiation. Additionally, NINJ1 plays a key role in membrane rupture during pyroptosis, a form of programmed cell death affecting granulocytes, while NLRP12 is involved in inflammasome formation and may contribute to pyroptosis through the PANosome [52]. Taken together, these findings suggest that the GAS7 cluster and its constituents could serve as valuable targets for further research into immune cell function. More broadly, these results indicate that vitamin D may exert temporally coordinated effects on diverse biological processes.

Interindividual variability in the response to vitamin D_3_ supplementation was another critical finding of this study. Based on differential gene expression analysis, the 25 VitDHiD participants were classified into two distinct groups, with 14 genes showing significantly higher expression in one group compared to the other. This variability suggests that individual-specific factors, such as genetic background, baseline vitamin D status, or circadian regulatory mechanisms, may influence the responsiveness of vitamin D target genes. Further genomic analysis revealed that all 14 genes distinguishing the two groups contained VDR-binding enhancers within 2 to 455 kb of their TSS, supporting the hypothesis that differential VDR-mediated regulation contributes to interindividual differences in gene expression. However, this aspect needs to be explored in more detail, since the in vitro THP-1 model may not reflect all VDR binding sites used in vivo.

The circadian in vivo vitamin D target genes, *AQP9*, *ASCL1* (acyl-CoA synthetase long chain family member 1), *IRS2* (insulin receptor substrate 2), *NAMPT*, *PFKFB3*, *PRKAG2* (protein kinase AMP-activated non-catalytic subunit gamma 2), *SLC2A3*, and *SLC6A6*, encode proteins with crucial roles in energy metabolism and cellular function. ASCL1 contributes to fatty acid metabolism; AQP9 functions as a glycerol and urea transporter; IRS2 mediates the downstream effects of insulin signaling; NAMPT is essential for cellular respiration, as it plays a key role in NAD^“^ biosynthesis; PFKFB3 regulates glycolysis; PRKAG2 is a subunit of AMPK (AMP-activated protein kinase), the master regulator of metabolism with circadian crosstalk capabilities [53]; while SLC2A3 facilitates glucose transport and SLC6A6 taurine transport. These findings highlight vitamin D’s evolutionarily ancient role in regulating energy metabolism [54], while also emphasizing the significance of key metabolic products, NAD^“^, glucose, and fatty acids, in circadian biology [55]. In recent years, the circadian aspect of health, particularly its connection to metabolism, has gained increasing attention, notably in the context of the so-called circadian syndrome [56]. This condition links metabolic syndrome to disruptions in the circadian rhythm.

This study has several limitations. Most notably, the relatively small cohort size of the VitDHiD study (25 individuals) limits the statistical power to detect subtle differences between chronotype groups. The limited power may, in fact, suggest that additional, as-yet-undetected differences exist between the clusters studied. Furthermore, the participants were primarily of Finnish origin, representing one of the most genetically homogenous populations worldwide, which may limit the generalizability of the findings. Another limitation is that the VitDHiD study was not originally designed with a circadian focus, and no data on participants’ biorhythms were collected. Finally, although the use of a monthly vitamin D_3_ bolus appears effective for identifying in vivo vitamin D target genes, the results should be validated using a regimen of daily, moderate vitamin D_3_ supplementation.

## 5. Conclusions

Overall, our findings emphasize the circadian nature of vitamin D target gene regulation and highlight interindividual variability in the transcriptional response to vitamin D_3_ supplementation. These insights have important implications for personalized vitamin D_3_ supplementation strategies, suggesting that optimal dosing regimens may need to consider both circadian biology and individual responsiveness to vitamin D. For instance, vitamin D has been linked to improved sleep quality [57], indicating that one of its few well-established health benefits may have a circadian component. However, there is currently no consensus on whether vitamin D_3_ supplementation is best taken at night or during the day, nor definitive evidence that vitamin D acts as a Zeitgeber. A Zeitgeber (German for “time giver”) is any external cue that helps regulate the human body’s circadian rhythms, such as light, mealtime, physical activity, social interactions, noise, or temperature changes. Future studies should further investigate the molecular mechanisms underlying these differences, including potential genetic and epigenetic factors that influence VDR activity and circadian gene expression. Additionally, further research is needed to determine whether vitamin D has Zeitgeber properties and how it may contribute to circadian entrainment. In conclusion, our findings reveal a strong and previously underappreciated link between vitamin D signaling and circadian biology in human PBMCs. The identification of distinct gene expression clusters and interindividual differences in vitamin D response further emphasize the need for a personalized approach to vitamin D_3_ supplementation, potentially incorporating circadian timing as a factor in optimizing its biological effects.

## Data Availability

Fastq files of the raw data of the VitDHiD study can be found at Gene Expression Omnibus (GEO) with accession number GSE260981.

## Supplementary Materials

The following Supporting Information can be downloaded at: https://www.mdpi.com/article/10.3390/nu17071204/s1, Figure S1: Seasonal Vitamin D Target Genes; Figure S2: Basal Expression and Inducibility of Circadian Vitamin D Target Genes; Figure S3: VDR-Binding Enhancers in the Genomic Regions of Ten Vitamin D Target Genes; Table S1: Vitamin D3-Induced Transcriptome of PBMCs In Vivo; Table S2: Differential Gene Regulation Analysis.

## Abbreviations

The following abbreviations are used in this manuscript:

1,25(OH)_2_D_3_	1α,25-dihydroxyvitamin D_3_
AMIGO2	adhesion molecule with Ig-like domain 2
AMPK	AMP-activated protein kinase
AQP9	aquaporin 9
ASCL1	acyl-CoA synthetase long chain family member 1
BCL	BCL transcription coactivator
C5AR1	complement C5a receptor 1
CCR7	C-C motif chemokine receptor 7
CD3G	CD3 gamma subunit of T-cell receptor complex
CD93	CD93 molecule
ChIP-seq	chromatin immunoprecipitation sequencing
CPM	counts per million
CPPED1	calcineurin-like phosphoesterase domain containing 1
CREB5	CAMP responsive element binding protein 5
CRISPLD2	cysteine-rich secretory protein LCCL domain containing 2
CSF2RB	colony stimulating factor 2 receptor subunit beta
CSF3R	colony stimulating factor 3 receptor
CSRNP1	cysteine- and serine-rich nuclear protein 1
DYSF	dysferlin
FAIRE-seq	formaldehyde-assisted isolation of regulatory elements followed by sequencing
FC	fold change
FCGR2A	Fc gamma receptor IIa
FDR	false discovery rate
FGR	FGR proto-oncogene, Src family tyrosine kinase
FOSL2	FOS-like 2, AP1 transcription factor subunit
GADD45B	growth arrest and DNA damage inducible beta
GAS7	growth arrest specific 7
IRAK3	interleukin 1 receptor-associated kinase 3
IRS2	insulin receptor substrate 2
JDP2	Jun dimerization protein 2
KLF11	KLF transcription factor 11
MAP3K7CL	MAP3K7 C-terminal like
MYC	MYC proto-oncogene, BHLH transcription factor
NAMPT	nicotinamide phosphoribosyltransferase
NDEL1	NudE neurodevelopment protein 1 like 1
NINJ1	ninjurin 1
NLRP12	NLR family pyrin domain containing 12
PADI2	peptidyl arginine deiminase
PBMC	peripheral blood mononuclear cell
PER1	period circadian regulator 1
PFKFB3	6-phosphofructo-2-kinase/fructose-2,6-biphosphatase 3
PILRA	paired immunoglobin-like type 2 receptor alpha
PRKAG2	protein kinase AMP-activated non-catalytic subunit gamma 2
RARA	retinoic acid receptor alpha
RNA-seq	RNA sequencing
RNF19B	ring finger protein 19B
SIK3	SIK family kinase 3
SIPA1L2	signal-induced proliferation-associated 1 like 2
SIRPB2	signal regulatory protein beta 2
SLC	solute carrier family
STX11	syntaxin 11
TAD	topologically associating domain
TIMP2	TIMP metallopeptidase inhibitor 2
TOM1	target of Myb1 membrane trafficking protein
TPCN2	two pore segment channel 2
TREM1	triggering receptor expressed on myeloid cells 1
TSC22D3	TSC22 domain family member 3
TSS	transcription start site
VDR	vitamin D receptor
WDFY3	WD repeat and FYVE domain containing 3
ZNF	zinc finger protein
