# Supplementary material for: Circadian Regulation of Vitamin D Target Genes Reveals a Network Shaped by Individual Responsiveness
Source: Nutrients. 2025 Mar 29;17(7):1204. doi: 10.3390/nu17071204 (PMC11990303; doi:10.3390/nu17071204)
Supplement: Supplementary file 1 [file nutrients-17-01204-s001.zip › Supplements/Supplementary legends.pdf]

Supplementary Data

Supplementary Tables

Table S1: Vitamin D_3-Induced Transcriptome of PBMCs *In Vivo*. RNA-seq analysis was performed on PBMCs isolated immediately before (d0) and 24 hours after (d1) a vitamin D_3 bolus supplementation. Across all 50 samples, 7,665 genes were expressed at CPM $\geq$ 10. Of these, 361 genes were significantly regulated (FDR $\leq$ 0.05) in response to vitamin D_3 supplementation (highlighted in green).

Table S2: Differential Gene Regulation Analysis. Differential gene expression analysis of 87 vitamin D target genes was performed using EdgeR, comparing individuals from groups 1 and 2 (**Fig. 3**). Genes with significantly different expression levels (FDR $\leq$ 0.1) are highlighted in green.

Supplementary Figures

Figure S1: Seasonal Vitamin D Target Genes. Gene/protein interaction network depicting the 18 seasonal genes that are also *in vivo* vitamin D targets, with connecting lines indicating known interactions.

Figure S2: Basal Expression and Inducibility of Circadian Vitamin D Target Genes. (**A**) Box plots displaying the range of basal expression levels (log scale) for the 87 circadian genes identified as *in vivo* vitamin D targets. (**B**) Box plots illustrating the inducibility (logFC) of these genes across the 25 individuals in the VitDHiD study. Genes are sorted by median expression level.

Figure S3: VDR-Binding Enhancers in the Genomic Regions of Ten Vitamin D Target Genes. The IGV browser was used to visualize ChIP-seq results for VDR (red) and FAIRE-seq data (blue) from THP-1 cells treated with solvent (0 hours) or 1,25(OH)$_2$D$_3$ (1,25D) for 2 and 24 hours. TSS regions and VDR-binding enhancers are shaded in grey. Peak tracks represent merged data from three biological replicates. Gene structures are depicted in blue, with vitamin D target genes highlighted in red. While genomic regions spanning 1 Mb upstream and downstream of each gene's TSS were analyzed, only areas relevant to 1,25(OH)$_2$D$_3$-dependent regulation are shown.
