# Supplementary figures and images for: Circadian Regulation of Vitamin D Target Genes Reveals a Network Shaped by Individual Responsiveness
Source: Nutrients. 2025 Mar 29;17(7):1204. doi: 10.3390/nu17071204 (PMC11990303; doi:10.3390/nu17071204)

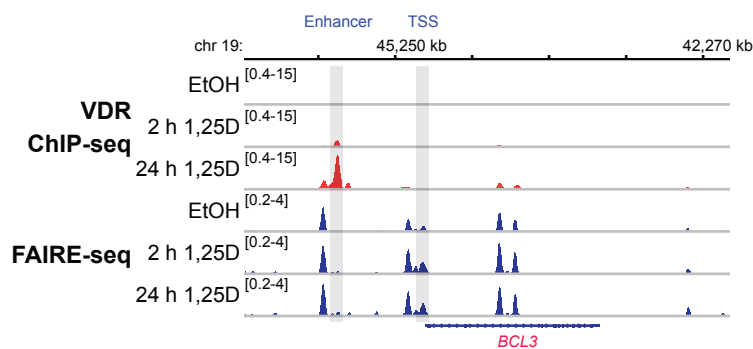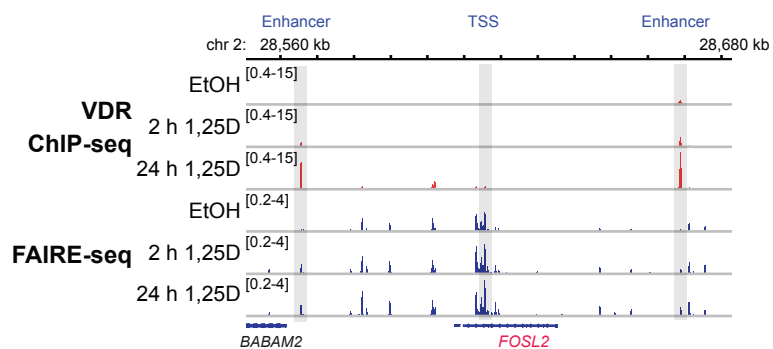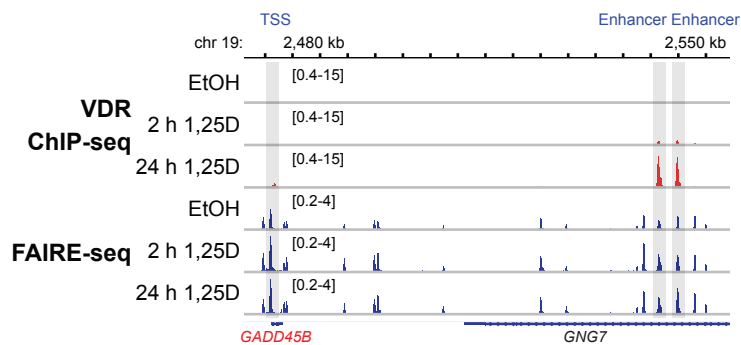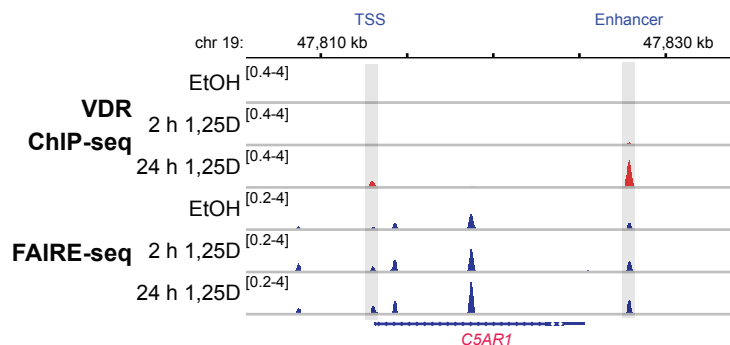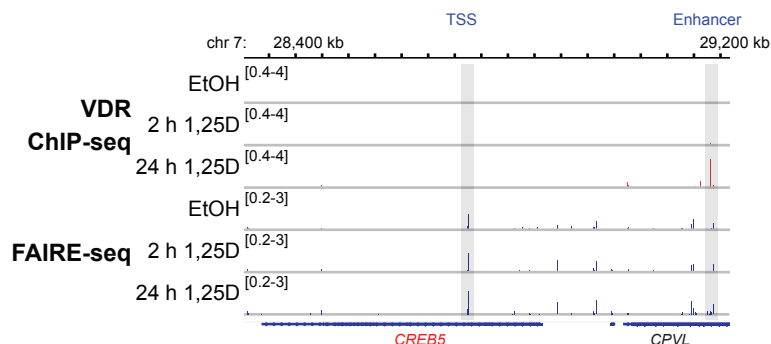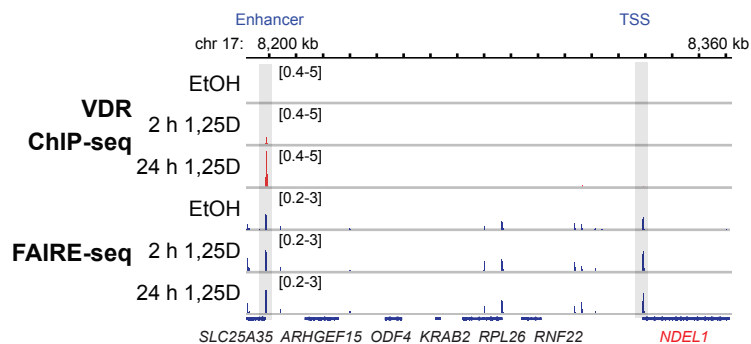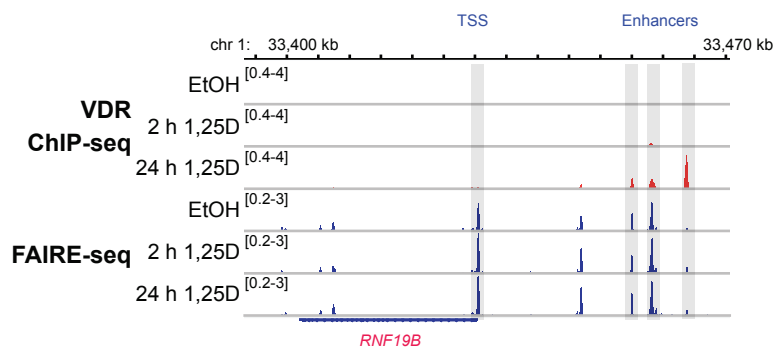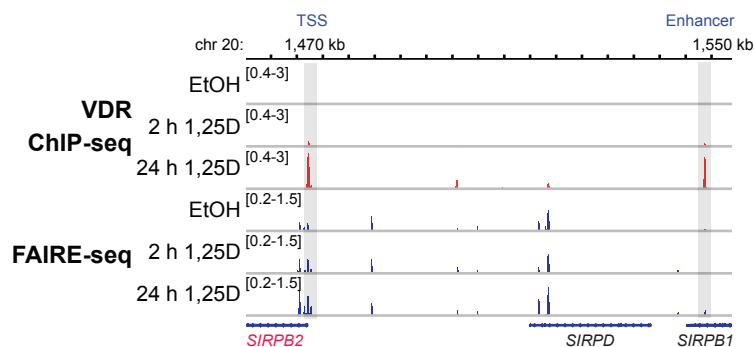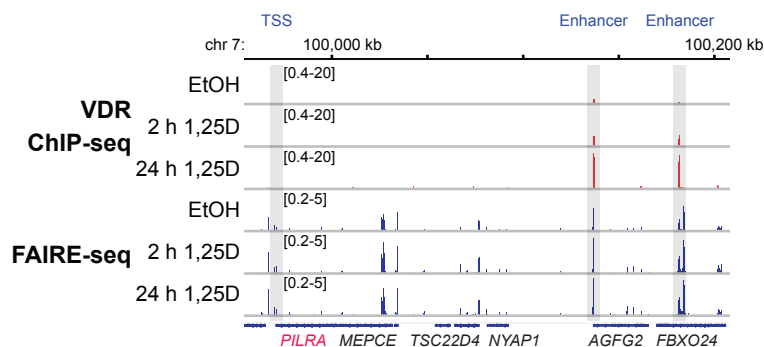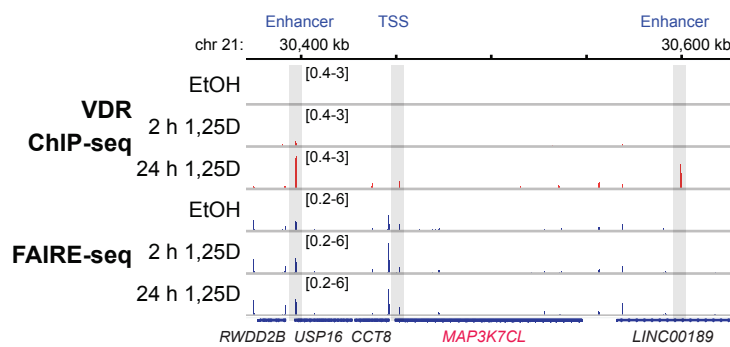

Supplement: Supplementary file 1 [file nutrients-17-01204-s001.zip › Supplements/Fig. S3.pdf]

**A**

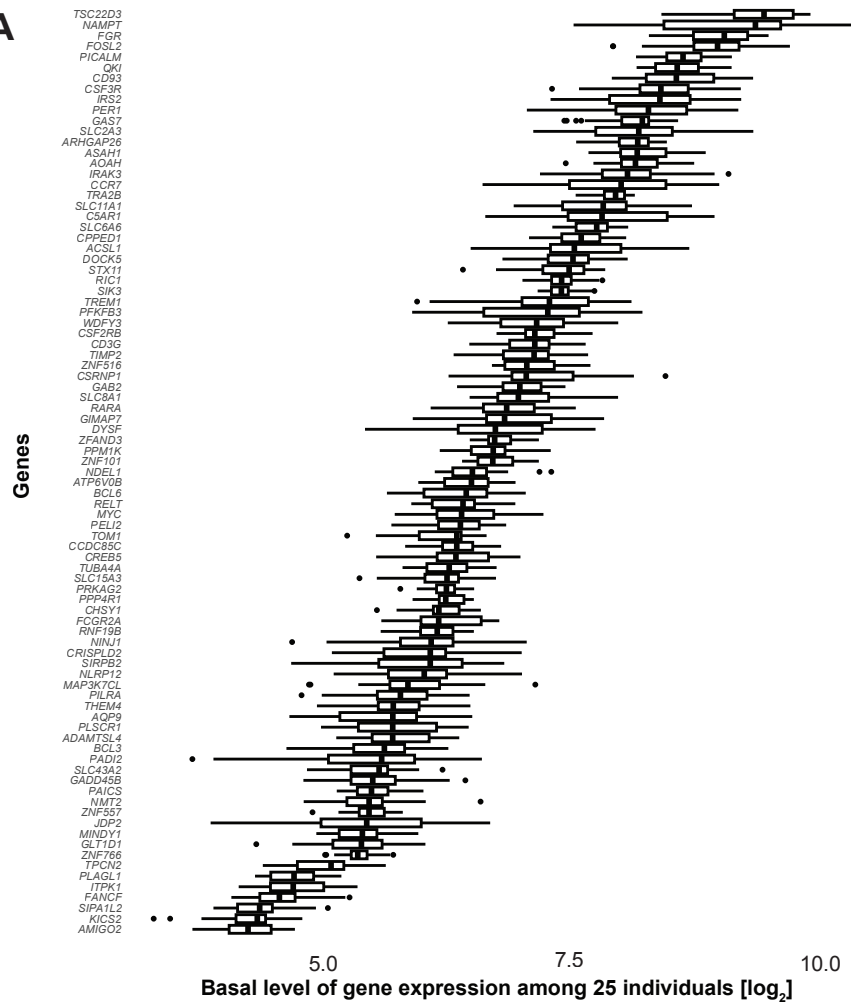

**B**

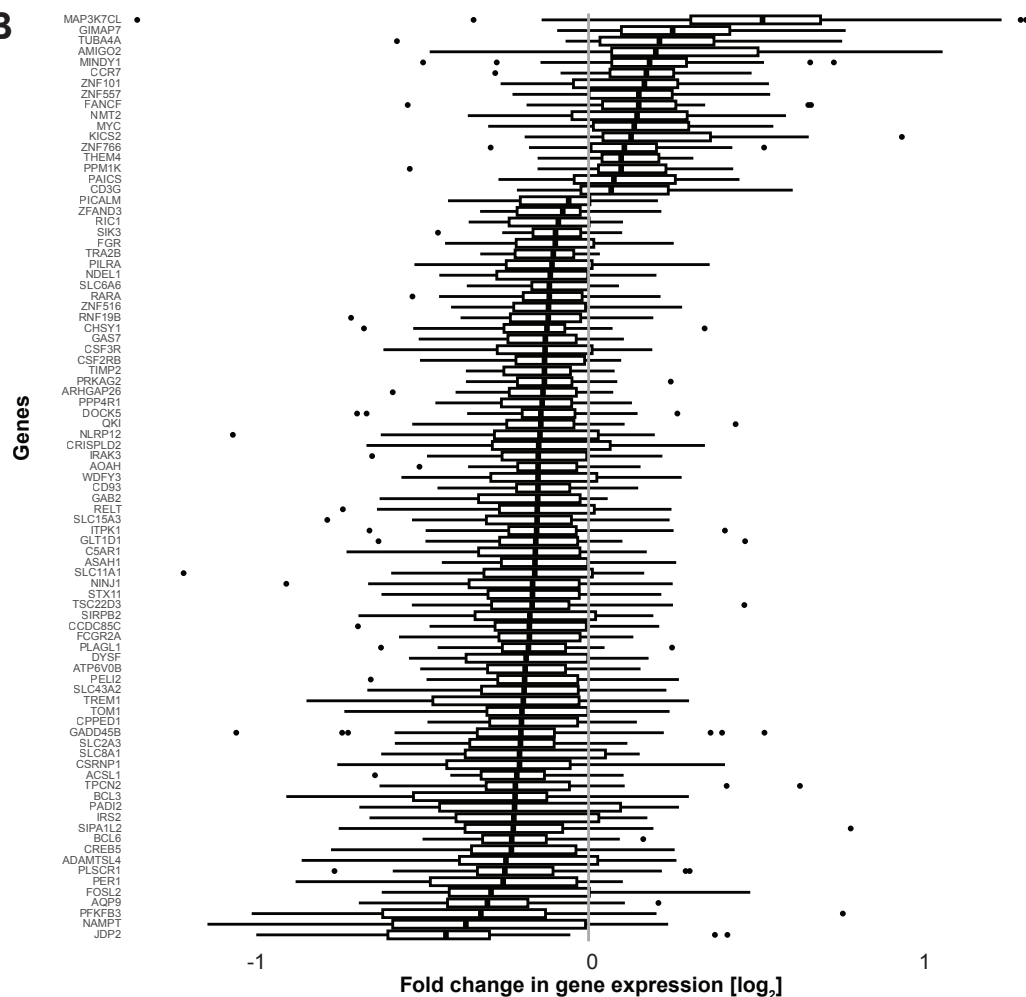

Supplement: Supplementary file 1 [file nutrients-17-01204-s001.zip › Supplements/Fig. S2.pdf]

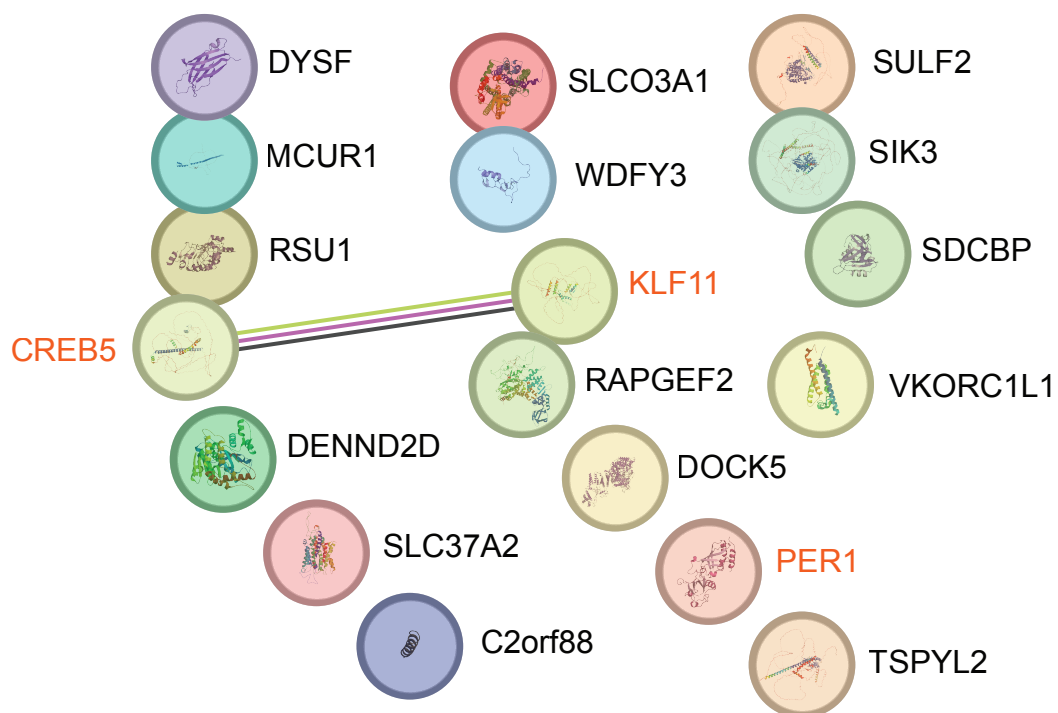

Supplement: Supplementary file 1 [file nutrients-17-01204-s001.zip › Supplements/Fig. S1.pdf]
